# Myogenic Precursor Cells Show Faster Activation and Enhanced Differentiation in a Male Mouse Model Selected for Advanced Endurance Exercise Performance

**DOI:** 10.3390/cells11061001

**Published:** 2022-03-16

**Authors:** Stefan Petkov, Julia Brenmoehl, Martina Langhammer, Andreas Hoeflich, Monika Röntgen

**Affiliations:** 1 Institute of Muscle Biology and Growth, Research Institute for Farm Animal Biology (FBN), Wilhelm-Stahl-Allee 2, 18196 Dummerstorf, Germany; petkov@fbn-dummerstorf.de; 2 Institute of Genome Biology, Research Institute for Farm Animal Biology (FBN), Wilhelm-Stahl-Allee 2, 18196 Dummerstorf, Germany; hoeflich@fbn-dummerstorf.de; 3 Lab Animal Facility, Institute of Genetics and Biometry, Research Institute for Farm Animal Biology (FBN), Wilhelm-Stahl-Allee 2, 18196 Dummerstorf, Germany; martina.langhammer@fbn-dummerstorf.de

**Keywords:** muscle stem cells, voluntary physical activity, proliferation, myogenic differentiation, Pax7, MyoD

## Abstract

Satellite cells (SATC), the most abundant skeletal muscle stem cells, play a main role in muscle plasticity, including the adaptive response following physical activity. Thus, we investigated how long-term phenotype selection of male mice for high running performance (Dummerstorf high Treadmill Performance; DUhTP) affects abundance, creatine kinase activity, myogenic marker expression (Pax7, MyoD), and functionality (growth kinetics, differentiation) of SATC and their progeny. SATC were isolated from sedentary male DUhTP and control (Dummerstorf Control; DUC) mice at days 12, 43, and 73 of life and after voluntary wheel running for three weeks (day 73). Marked line differences occur at days 43 and 73 (after activity). At both ages, analysis of SATC growth via xCELLigence system revealed faster activation accompanied by a higher proliferation rate and lower proportion of Pax7+ cells in DUhTP mice, indicating reduced reserve cell formation and faster transition into differentiation. Cultures from sedentary DUhTP mice contain an elevated proportion of actively proliferating Pax7+/MyoD+ cells and have a higher fusion index leading to the formation of more large and very large myotubes at day 43. This robust hypertrophic response occurs without any functional load in the donor mice. Thus, our selection model seems to recruit myogenic precursor cells/SATC with a lower activation threshold that respond more rapidly to external stimuli and are more primed for differentiation at the expense of more primitive cells.

## 1. Introduction

Skeletal muscle is mainly composed of multinucleated myofibers. In mice, as in other species, the number of myofibers is fixed at birth, and postnatal muscle growth is achieved by hypertrophic fiber growth [1]. This process is most intense during the first three weeks of life and contributes to half of the seven- to eight-fold increase in body weight that occurs during this period in mice [2]. Muscle progenitor cells, so-called satellite cells (SATC), are essentially involved in this most dynamic phase of postnatal growth [3]. After this period, the adult number of SATC and myonuclei is already established in mice [3]. The size and composition of the produced SATC pool greatly affect postnatal muscle growth, maintenance, and repair [4]. Therefore, the number of myogenic precursor cells (MPC)/SATC and their molecular/functional properties play a significant role in muscle development, plasticity, and regeneration [4].

SATC are heterogeneous, and, as in other species, two distinct SATC populations have been identified in mice, including committed progenitors responsible for muscle growth and routine maintenance (≈90%) and reserve SATC (≈10%) [5,6,7]. The proportion of SATC decreased with age, from about 30% at birth to 13 and 3% at postnatal weeks two and seventeen [8,9,10]. During the first three postnatal weeks, however, a high percentage (80%) of these SATC is proliferating [1,11], whereas, in the adult, most SATC (97–99%, [12]) are quiescent but poised to activation. SATC determination, functional stages, and fate are regulated by expressing several characteristic myogenic regulatory factors that also participate in cell cycle regulation, hypertrophy, and fiber type determination [13].

For juvenile myogenic progenitors (until about day 21 of life), paired box protein 7 (Pax7) expression is critical for survival, maintenance of their expansive and myogenic capacity, and transition into the adult, quiescent state [14,15,16]. The latter process achieves the regenerative capacity of muscles [16]. During quiescence, Pax7 and myogenic factor 5 (Myf5) are expressed, whereas myoblast determination protein 1 (MyoD) is induced in activated/proliferating myoblasts [17,18,19]. Under in vivo conditions, juvenile SATC progenitors of mice are actively proliferating and contribute to the extensive myofiber growth by the net addition of myonuclei. Myoblasts expressing both Pax7 and MyoD form the largest group in the proliferating population [20].

Myf5 and/or MyoD expressing cells can return to quiescence to maintain the SATC pool. Transition into differentiation is initiated by downregulation of Pax7 and increased expression of MyoD, leading to induction of myogenin (MyoG) (early differentiation; myocytes). In vitro, differentiation can be induced by serum starvation and comprises a sequence of highly ordered events: after myogenin expression (mark cells as irreversibly committed to differentiation), permanent cell cycle withdrawal is induced by p21 [21], followed by the production of various contractile proteins including sarcomeric myosin heavy chain (MyHC) proteins (phenotypic differentiation) and fusion [17,22,23]. Fusion consists of two distinct stages, namely, initial myoblast-myoblast fusion to establish nascent myotubes and subsequent myoblast-myotube fusion to increase myotube size [24].

Muscle SATC are essential for muscle repair/regeneration, but their involvement in muscle atrophy and hypertrophy is not fully understood [25]. SATC are known to be activated in muscles to proliferate during muscle hypertrophy and to be incorporated into myofibers, increasing the number of myonuclei. This “myonuclei domain theory” is advocated by many researchers and states that increasing myofiber size requires new myonuclei from SATC to maintain the ratio of myonuclei to cytoplasm, thereby increasing muscle hypertrophy and weight [26,27]. Interestingly, it has been shown in a SATC-depleted mouse model (Pax7-DTA) [20] that SATC-independent muscle fiber hypertrophy is possible [28] without an increase in the number of myonuclei [26,29].

Besides intrinsic signals, skeletal muscle and SATC functionality/phenotype are affected by environmental stimuli such as nutrients, growth factors, injury, and muscle use, e.g., physical exercise [30,31,32]. Physical activity is critical for muscle mass development and maintenance and, thus, its functionality. By studying SATC in vivo, a positive effect of physical exercise on these cells was demonstrated. Voluntary running wheel (RW) activity for six or eight weeks leads to increased SATC numbers, identified by Pax7 staining, and improves the differentiation capacity in rats’ plantaris [33] or vastus lateralis and medial gastrocnemius muscles [34]. Kurosaka et al. could also show a positive correlation between the percentage of SATC and the running distance [33]. Also, enhanced proliferation in murine musculus plantaris and musculus soleus was reported in response to four-week RW activity [35]. The use of genetically labeled SATC impressively visualized in vivo that more SATC fused with fibers in the musculus soleus, musculus plantaris, and musculus gastrocnemius after eight weeks of voluntary RW activity than under sedentary conditions [36]. Long-term moderate-intensity treadmill running (13 weeks) increases the number of SATC per myofiber in the musculus gastrocnemius of female and male rats at the age of 3.5 and 15 months, respectively [37]. Studies on proliferation and the percentage distribution of quiescent, proliferating, and activated SATC isolated from the limb muscles of sedentary mice of different ages or after voluntary activity do not exist to our knowledge.

An excellent model to study the features of SATC in mice is provided by the non-inbred Dummerstorf marathon mouse model DUhTP and its associated control line. Due to paternal phenotype selection over 140 generations, the line DUhTP has a genetically fixed high running ability without previous training [38,39] accompanied by diminished voluntary running wheel activity [40]. A series of experiments [41,42,43] revealed marked modifications in the energy metabolism in liver and subcutaneous adipose tissue of these DUhTP mice, specifically in fat accumulation and mobilization. In sedentary conditions, they accumulate high amounts of body fat, while voluntary activity in RW completely abolished the obese phenotype, indicating the physiological relevance of prominent mitochondrial oxidation for superior endurance exercise performance of DUhTP mice [42]. Effects of long-term selection for high treadmill performance on MPC/SATC abundance, functional properties and fate, and their role in the muscular adaptive response to exercise have never been investigated in DUhTP mice.

SATC number and, thus, bioavailability is a main determinant of postnatal muscle growth, muscle homeostasis by replenishment of aging myonuclei [4], and for muscle regeneration after injuries. We assume that the number of MPC/SATC is higher in DUhTP mice and determined SATC abundance (as SATC number per gram muscle) to identify possible age-dependent and/or line-dependent differences and the effect of voluntary activity.

Regarding SATC functionality, we hypothesize that MPC from DUhTP mice show an increased ability to proliferate and differentiate, thereby contributing to their superior forced running capacity. To include different stages (juvenile, proliferating; maturation, transition to quiescence; adult, quiescent) [3,44] of postnatal SATC development, cells were isolated from the limb muscles of male mice of different ages (12, 43, 73 d).

## 2. Materials and Methods

### 2.1. Mouse Line Establishment

The Dummerstorf mouse lines have been initially generated by systematic crossbreeding of four inbred (CBA/Bln, AB/Bln, C57BL/Bln, and XVII/Bln) and four outbred lines (NMRI orig., Han:NMRI, CFW, and CF1) to obtain a mouse strain with a broad genetic background [45]. From this genetic pool (FztDU), the control line Dummerstorf Control mouse line (DUC) has been established by random mating. The mouse line DUhTP used in the present study had been generated from the same base population by paternal selection for high treadmill performance for 140 generations, four generations per year [38]. Therefore, submaximal high treadmill performance was determined after mating in around 77-day-old male mice, enhancing the selection process’s speed. Treadmill performance was determined by a singular submaximal test (0% incline). According to the protocol, mice performed first an initial run for 30 m at a speed of 12 m/min (≙150 s). Then, after stopping the treadmill for 1 min and a second run for 20 m at 12 m/min (≙100 s), the treadmill speed was gradually increased up to a maximum speed of 38 m/min. The stepwise increases consisted of running distances of 50 m each at 22 m/min (≙136 s), 26 m/min (≙115 s), 30 m/min (≙100 s), 34 m/min (≙88 s), and 36 m/min (≙83 s)). Thus, the terminal velocity was reached after 300 m (≙832 s). The test was terminated when the mice repeatedly rested on the stimulation device at the end of the treadmill. The endurance fitness was recorded as running distance in meters on a treadmill. The offspring of the males with the highest running performance were selected as parents for the next generation. By this strategy, a direct effect of the physical performance itself on breeding performance was excluded. Instead, a genetically fixed higher forced running ability without previous training was generated. Both lines, DUhTP and DUC, were maintained by minimizing inbreeding. For this purpose, the control line was bred with 125–200 breeding pairs and the DUhTP line with 60–100 breeding pairs per generation during the whole selection period.

Compared to the animals at the selection start, a selection success of 400% was achieved in DUhTP mice. DUhTP mice run longer than DUC controls, covering a 3.8-fold longer running distance than the unselected control line (DUhTP: 5832.1 ± 838.2 m, DUC: 1537.3 ± 200.5 m). Both lines are non-inbred strains characterized by high variability between individuals of one generation or different generations.

### 2.2. Animals and Study Design

All in vivo experiments were performed at the Research Institute of Farm Animal Biology (FBN) in Dummerstorf. Animal husbandry in the Laboratory for Innovative Farm Animal Models (LIN) of the FBN and slaughter followed the guidelines of the Animal Care Committee of the State Mecklenburg-Western Pomerania, Germany, based on the German Law of Animal Protection (Animal Welfare Act; TierSchG), and were approved by our internal institutional review board. As animals were not manipulated before slaughter, no animal experiment was conducted according to the German Animal Welfare Act.

**Figure 1:**
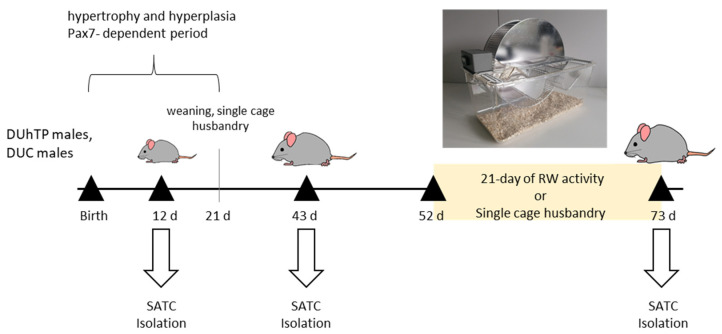
Experimental design. For the isolation of primary myogenic precursor cells/satellite cells from whole limb muscles, male DUC and DUhTP mice of different ages (12, 43, and 73 days) were used. Seventy-three-day-old mice either had access to a running wheel (RW) with a wheel counter (Tecniplast, Hohenpeißenberg, Germany) from day 52 of age or were kept entirely under sedentary conditions.

Mice of the lines DUhTP and DUC were kept in H-Temp Polysulfon cages (floor area: 370 cm^2^; Eurostandard Type II, Tecniplast, Hohenpeißenberg, Germany) in specified pathogen-free conditions and provided with fresh water and autoclaved Ssniff^®^ M-Z feed (Ssniff-Spezialdiäten GmbH, Soest, Germany) ad libitum. After weaning, males of both lines were randomly assigned to different groups and kept in single cages until days 43 and 73 of life, respectively (Figure 1). One half of the latter group had access to an RW in the cage (activity wheel for rats; Tecniplast, Hohenpeißenberg, Germany) from 52 days of age, which they could use voluntarily for three weeks, while the second half was kept as sedentary controls in cages without RW. Voluntary physical activity was registered daily by a wheel counter that recorded all quarter rotations (Figure 1). Based on the RW activity (quarter rounds per day), individual activity was calculated, where one complete revolution of the wheel (diameter = 33.4 cm) corresponded to a running distance of 1 m.

On days 12, 43, or 73, the mice were weighed, sacrificed, and total limb muscle masses were determined after removing surrounding and intermuscular fat. A total of 94 DUC animals (12 d DUC: 20, 43 d DUC: 22, 73 d DUC: 27, 73 d DUC act: 25) and 79 DUhTP mice (12 d DUhTP: 16, 43 d DUhTP: 25, 73 d DUhTP: 22, 73 d DUhTP act: 16) were included in the experiment and selected for different trials. The mice were from a total of six generations (DUC: 190–193, 195–196; DUhTP: 145–148, 150–151) between May 2018 and November 2019, with three to twelve animals per age or activity group.

### 2.3. Satellite Cell Isolation and Culture

Total limb muscles (musculus quadriceps femoris, musculus biceps femoris, musculus triceps brachii, extensors, and flexors of the elbow) free of fat and tendons were used for MPC/SATC isolation (Figure 1). Muscles were cut into small pieces, transferred to an enzyme mix containing 2% collagenase Type I (Collagenase from *Clostridium histolyticum*, Sigma-Aldrich, St. Louis, MO, USA) and 2% dispase (Dispase II from *Bacillus polymoxa*, Roche, Mannheim, Germany), and incubated three times for 10 min with intervening mixing/shearing at 37 °C. The suspension was filtered through a 70 µm filter and then incubated at room temperature with a cocktail of monoclonal antibodies against non-target cells (Satellite Cell isolation kit mouse, Miltenyi Biotec, Bergisch Gladbach, Germany) conjugated to MACS^®^ MicroBeads. After 30 min, the cell suspension was filtered through a 50 µm filter. The magnetically labeled non-target cells were depleted by retaining them within the MACS Column in the magnetic field of a MACS Separator, while the unlabeled SATC passed through the column. Cell size and viability were measured (Countess Automatic cell counter, Thermo Fisher Scientific, MA, USA), and the number of live cells was calculated as cells per gram of muscle (10^5^). After isolation, cells have spent 40 min in untreated and uncoated dishes (Corning Incorporated, Durham, NC, USA) with growth medium (HAMS F10, 20% Fetal bovine serum (FBS), 2% Penicillin-Streptomycin solution, 1% Amphotericin; PAN Biotech, Aidenbach, Germany) in order to remove fibroblasts (pre-plating). Fibroblasts are heavier than SATC and attach faster to the dish bottom. After pre-plating, floating MPC/SATC were harvested, seeded in collagen type I (Greiner Bio-one, Monroe, NC, USA) coated dishes, and cultured in growth medium under a humidified atmosphere with 5% CO_2_ at 37 °C. Twenty-four hours after seeding, the cells were washed with Dulbecco’s phosphate-buffered saline (DPBS, PAN Biotech). A fresh growth medium was added, and cells were cultured for three days before renewing the medium. Bacterial and fungal contamination of cells was excluded via inoculation of CASO Bouillon Tryptic Soy Broth and Thioglycolate medium EP (Becton Dickinson, Heidelberg, Germany). For passaging, cultured MPC/SATC were detached by using Accutase cell detachment solution (PAN Biotech), and the reaction was stopped by adding growth media. After centrifugation (5 min, 453 g, 22 °C), cells were re-suspended in the growth medium, and the cell number was determined. The cell morphology was evaluated using a photonic microscope Carl Zeiss Primovert Ser. No. 38420210210.

After the first passage, part of the cells from single mice was used to determine the myogenic markers Pax7 and MyoD and to measure creatine kinase activity (see below). The numbers of animals used per experiment are indicated individually. The magnetic cell separation approach used for SATC isolation in the present study led to a strong 46-fold enrichment of this cell population. Nevertheless, as a control of the myogenic origin of the cells, differentiation assays (see below) were also performed, while the rest of the cells were frozen and stored until making cell pools for further experiments (shown in this manuscript: kinetic growth curves; xCELLigence system, fusion index, areas covered by myotubes, myofiber number, and size distribution). A mixture consisting of 50% growth medium, 30% FBS, and 20% dimethyl sulfoxide (DMSO; Carl Roth, Karlsruhe, Germany) was used as the freezing medium.

After sampling cells over four to five generations, they were thawed to create cell pools. Cells of one age group but from different generations were pooled to obtain 200,000 cells per vial. Care was taken to ensure a similar proportion of cells from individual animals per pool. Therefore, pools consisted of a minimum of three and a maximum of nine different mice. Cell pools were grown to get a maximum of cell yield and, after that, finally seeded for the corresponding assay (e.g., xCELLigence, differentiation) or frozen for biochemical or metabolic studies (results are not part of this manuscript). The numbers of pools and the numbers of individual mice are thus indicated individually.

### 2.4. xCELLigence

In order to compare the functionality of MPC/SATC isolated from male DUC and DUhTP mice, growth kinetics, adhesion, and proliferation properties were continuously recorded over 72 h using the xCELLigence system (RTCA-SP, ACEA Biosciences Inc., San Diego, CA, USA), which measures impedance changes caused by the attachment and spreading of cells and calculates them as the dimensionless Cell Index (CI).

**Figure 2:**
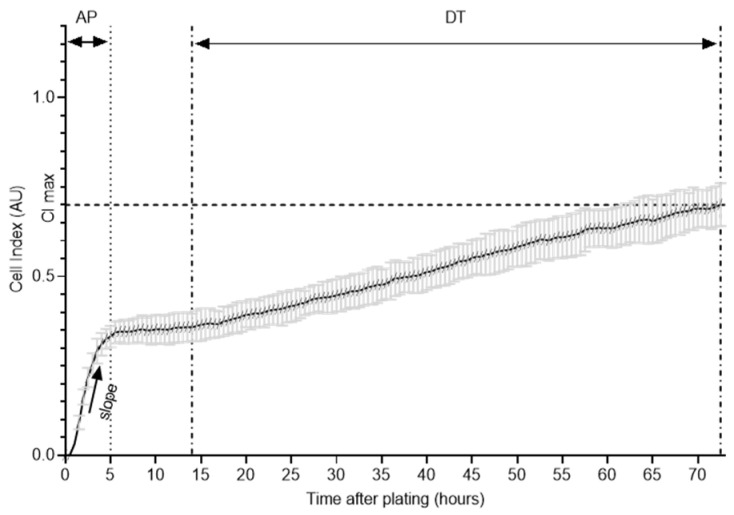
Schematic presentation of xCELLigence system experiment. The growth curves were analyzed within 72 h for slope during the adhesion period (AP), cell index (CI max), and doubling time (DT) during the logarithmic growth phase.

Therefore, forty thousand cells per well in quadruplicates were used in a 96-well-plate to assess quantitative adhesion and proliferation capabilities. The parameters slope (expressing the adhesion kinetics; AP), maximal cell index (highest CI value, usually associated with the stationary growth phase, here: end point value of the CI; CImax), and doubling time (DT, a measure of proliferation rate) were used and are shown exemplarily in Figure 2 [46,47].

More details on the xCELLigence technology can be found in Atienza et al. (2005) or Ke et al. (2011).

### 2.5. Immunofluorescence Staining for Myogenic Markers Pax7 and MyoD

MPC/SATC originating from single animals or cell pools were seeded at a density of 50,000 per well on a collagen-coated 24-well plate and cultured in the growth medium with 20% FBS (see Section 2.3) for 24 h. The cells were fixed with 4% Paraformaldehyde (PFA), then permeabilized with 0.1% Triton X100 in DPBS, and blocked with 5% Horse serum (HS, PAN Biotech) at room temperature. The primary anti-MyoD antibody (rabbit, Thermofisher Scientific) was 1:250 diluted in undiluted Hybridoma Mouse anti-Pax7 antibody (Developmental Studies Hybridoma Bank). Cells were incubated with both primary antibodies overnight at 4 °C. The next day, cells were washed with DPBS to remove unbounded primary antibodies and incubated for 45 min at room temperature with the secondary antibodies, diluted 1:1000 in 5% HS in DPBS (MyoD: Alexa 488 donkey anti-rabbit, Pax7: Alexa 546 goat anti-mouse; Thermo Fisher Scientific).

Pictures of the cells were taken with a fluorescent microscope Nikon Diaphot 300 (Nikon Corporation, Tokyo, Japan). To determine the total cell number, six pictures per sample, randomly chosen, were used. The software Photoshop CS6 (Adobe Photoshop Version: 13.0.1, Adobe Inc., San Jose, CA, USA) was used to create the overlay of pictures to adjust the brightness and contrast. Software Image J 2.0.0 Java 1.6 (National Institutes of Health (NIH), Bethesda, MD, USA) was used for quantitative analysis. While the total number of nuclei was counted automatically, nuclei, positive for Pax7 (Pax7+/MyoD−), MyoD (Pax7−/MyoD+), or both (Pax7+/MyoD+), were counted manually.

### 2.6. Differentiation of SATC

For myogenic differentiation, cells from single animals (passage one) or cell pools were seeded in Matrigel Growth Factor Reduced Basement Membrane Matrix (diluted 1:50, Corning) coated 24-well plates (50,000 cells/well) and cultured in growth medium. When cells started to show signs of spontaneous differentiation, differentiation was induced. After removing the growth medium, cells were washed with 37 °C DPBS, and the differentiation medium was added (PAN Biotech, DMEM with high glucose, 4.5 g/L, 5% HS, 2% Penicillin-Streptomycin). Experiments were performed 72 h after induction.

### 2.7. Immunofluorescence for Myosin Heavy Chain and Determination of the Fusion Index

For myosin heavy chain (MHC) staining, cells were fixed with 4% PFA and permeabilized with 0.1% TritonX100 in PBS. After blocking with 5% HS for 1 h, myotubes were incubated overnight with a primary mouse anti-skeletal myosin antibody (MF20 hybridoma) in DPBS. Subsequently, primary antibodies were removed by washing with DPBS. Then, myotubes were incubated with an Alexa 488-conjugated rabbit anti-mouse secondary antibody (Life Technologies, MA, USA), diluted 1:1000, and stained with DAPI (Sigma Aldrich). For imaging, the Nikon Diaphot 300 microscope with 10× magnification was used.

The pictures’ overlay was created using the software Photoshop CS6 (Version CS6, Adobe Inc., CA, USA); brightness and contrast were adjusted to the same degree in every sample group. MHC positive myotubes containing ≥2 nuclei were encircled to quantify the myotube area using the Software Image J 2.0.0 Java 1.6 (NIH). The total number of cell nuclei was counted automatically. For each experiment, six random sections were analyzed.

Micrographs of the MHC-stained cells were also used to determine the fusion index, according to Miersch et al. [48]. The number of nuclei in the fused multinucleated cells (≥2 nuclei) was divided by the total number of visible nuclei for six randomly chosen pictures per sample and multiplied by 100 to calculate the fusion index.

### 2.8. Creatine Kinase (CK) Measurement

Frozen MPC/SATC were thawed and differentiated for three days before CK activity was determined using the CK-NAH hit kit (IFCC method, BIOMED Labordiagnostik GmbH, City, Germany) according to the manufacturer’s instructions.

### 2.9. Statistical Analyses

Data from xCELLigence system are presented as means ± standard deviation (SD), data concerning the distribution of myotube area and Pax7 and/or MyoD expressing cells are demonstrated as stacked columns, whereas all other data are shown as Box-Whisker plots with the median. Statistical analyses were performed using Graph Pad Prism (Version 9.2, Graph Pad Software, San Diego, CA, USA) and SigmaPlot 14.0 (Systat Software Inc, Chicago, IL, USA). Data from cell isolation parameters, body weight, muscle mass, recording of kinetic growth curves (xCELLigence system experiment), and immunostaining of myogenic markers were tested with one-way ANOVA, Tukey’s multiple comparison test. Data from differentiation assays, quantification of fusion index, sedentary live cell yield comparison, and creatine kinase measurements were tested with one-way ANOVA, Tukey’s multiple comparison test and unpaired *t*-test with Welch’s corrections. Statistical differences were considered single factorial between the lines at the same age (line-specific effects) or within each mouse line at different ages (age-related effects) or physical activity status (physical activity-related effects). A *p*-value of <0.05 was considered to be statistically significant.

## 3. Results

### 3.1. Voluntary Running Performance, Body and Muscle Weight, and Satellite Cell Yield

The voluntary activity level of DUC mice was 2.5-fold higher than that of DUhTP mice (DUC: 5684 ± 2694 m (*n* = 17), DUhTP: 2205 ± 1279 m (*n* = 16); *p* < 0.001).

**Figure 3:**
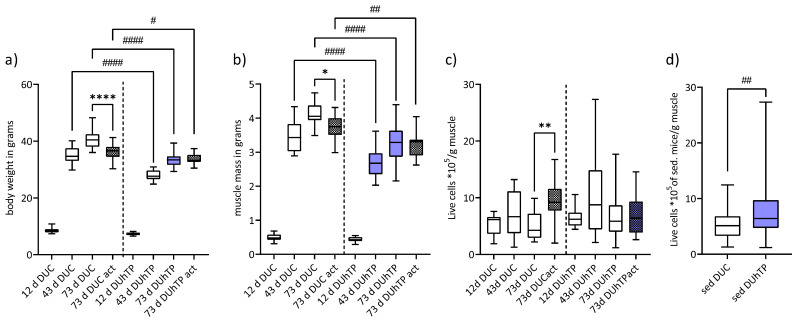
(**a**) Body weight and (**b**) muscle mass of all limbs of 12-, 43-, and 73-day-old male DUC (white bars) and DUhTP mice (blue bars), with older mice either sedentary or active in a running wheel (act). A total of 85 DUC (12 d: 20, 43 d: 22, 73 d: 26, 73 d act: 17) and 72 DUhTP mice (12 d: 16, 43 d: 18, 73 d: 22, 73 d act: 16) were included in the analyses. Satellite cells were isolated from whole limb muscles, and (**c**–**d**) the cell yield per gram of muscle was considered (**c**) for each age (DUC 12 d: 16, 43 d: 22, 73 d: 22, 73 d act: 17 mice; DUhTP 12 d: 16, 43 d: 18, 73 d: 22, 73 d act: 16 mice) and activity group or (**d**) holistically from all sedentary (sed) mice of a mouse line (DUC: *n* = 68 mice; DUhTP: *n* = 56 mice). Results are shown as means and SD, and statistical analyses were performed using one-way ANOVA (**a**–**c**) or unpaired *t*-test with Welch’s correction. Significant line-specific differences (DUC versus DUhTP mice) are marked by hashtags (^#^ *p* < 0.05; ^##^ *p* < 0.01; ^####^ *p* < 0.0001), while activity-induced differences (act versus sedentary) within the mouse lines are indicated by asterisks (* *p* < 0.05; ** *p* < 0.01; **** *p* < 0.0001).

From day 43 on, DUhTP mice had significantly lower body weight and muscle mass than DUC mice (Figure 3a,b). Since the body and muscle weights on day 12 did not differ significantly between the two lines, the DUC phenotypically showed a significantly higher growth capacity by about 30% than the DUhTP line until 43 days of age. After that, there was no difference in growth intensity. In response to voluntary physical activity, bodyand muscle mass were exclusively reduced in DUC mice (*p* < 0.05).

SATC yield obtained from limb muscles was similar in male DUC and DUhTP mice at different ages (Figure 3c). However, a generally higher SATC yield isolated from the muscles of sedentary DUhTP mice was observed compared to sedentary controls (Figure 3d; *p* < 0.005). Furthermore, a strong activity-induced increase in total cell yield was observed in DUC mice (Figure 3c; *p* < 0.01).

### 3.2. Growth Kinetics of Isolated Satellite Cells

**Figure 4:**
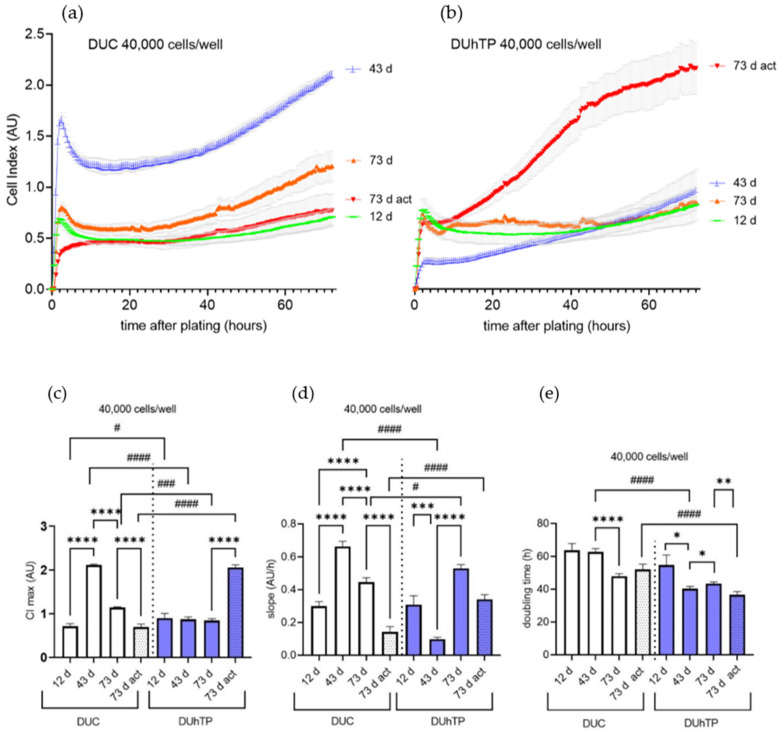
Cultured pools of MPC/SATC from male DUC (left and in white; pools (*n* = 3–4) represent: 12 d: 6 mice, 43 d: 16 mice, 73 d: 16 mice, 73 d act: 12 mice) and DUhTP animals (right and in blue; pools (*n* = 3) represent: 43 d: 9 mice, 12 d, 73 d, and 73 d act: 8 mice) were derived from sedentary animals at the age of 12, 43, 73 days (**d**) and physically active (act) animals at the age of 73 days. The impedance-based xCELLigence system was used for real-time monitoring of adhesion and proliferation of SATC populations seeded at densities of 40,000 cells per well. Kinetic growth curves (**a**,**b**) of SATC were recorded for 72 h, and (**c**) the maximum cell index (CI max), (**d**) the slope during the adhesion period, and (**e**) doubling time (DT) were analyzed. DT was obtained during the logarithmic growth phase, while the slope was calculated (ΔCI/Δtime) during the adhesion period. The data are shown in histograms as means and SD. Significant line-specific differences (DUC vs. DUhTP mice) are separated by hashtags (^#^ *p* < 0.05, ^###^ *p* < 0.001, ^####^ *p* < 0.0001), while significant age-related or activity-related differences within each line are separated by asterisks (* *p* < 0.05, ** *p* < 0.01, *** *p* < 0.001, **** *p* < 0.0001).

The isolated MPC/SATC were analyzed for their characteristic growth profile, typified by unique morphology, attachment, and proliferative behavior [49], using the xCELLigence system. Continuous real-time growth curves of myoblasts from male DUC and DUhTP mice were recorded over 72 h and are shown in Figure 4a,b, respectively. Kinetic CI traces from myoblasts of all DUC ages were characterized by a rapid adhesion followed by a distinct lag phase and a period of logarithmic growth. No plateau was reached during the measurements. CI curves of myoblasts from 12-day-old and sedentary 73-day-old DUC mice (Figure 4a; green and orange lines) were similar to those of coeval DUhTP mice (Figure 4b; green and orange lines). However, clear differences between lines were found at day 43 (blue lines) and when mice had performed voluntary activity (red lines). Typically, shortening of the lag phase and faster entry into logarithmic growth were observed when myoblasts from DUhTP mice were used (Figure 4b). The ability to proliferate (Figure 4c) and attach (Figure 4d) was clearly higher in myoblast from 43-day-old DUC mice (CImax 2.12 ± 0.02 AU, slope 0.70 ± 0.07 AU/h) compared to cells from 12-day-old (CImax 0.7 ± 0.6 AU, slope 0.30 ± 0.03 AU/h) and 73-day-old (CImax 1.15 ± 0.01 AU, slope 0.45 ± 0.03 AU/h) DUC mice (all *p* < 0.0001) or from myoblasts of 43-day-old DUhTP mice (CI 0.88 ± 0.06 AU, slope 0.10 ± 0.03 AU/h, *p* < 0.0001). DT was slower in 43-day-old DUC than 73-day-old DUC cells (*p* < 0.0001; Figure 4e). In contrast, cells from 43-days-old DUhTP mice had a faster DT than those from 12- or 73-day-old DUhTP mice (*p* < 0.05).

Compared with sedentary 73-day-old mice, voluntary activity led to decreased (*p* < 0.0001) CImax (0.70 ± 0.07 AU) and slope (0.14 ± 0.03 AU/h) in DUC cells, whether CImax increased in DUhTP cells (CImax: 2.10 ± 0.06 vs. 0.84 ± 0.04 AU; *p* < 0.0001). In proliferating cultures from active mice, CImax and slope were higher (*p* < 0.0001) in DUhTP than the DUC line. In addition, the DT was not affected by physical activity in DUC mice (control: 52.07 ± 3.3 h, act: 56.6 ± 2.7 h), but significantly reduced in DUhTP mice (control: 43.7 ± 2.6 h, act: 36.5 ± 2.01 h, *p* < 0.01). Considering both lines, myoblasts from 43-day-old sedentary and 73-day-old active DUhTP mice were characterized by a shorter DT than DUC cells from the same groups (*p* < 0.0001; Figure 4e).

### 3.3. Pax7 and MyoD Expression in MPC/SATC Cultures from Male DUC and DUhTP Mice

**Figure 5:**
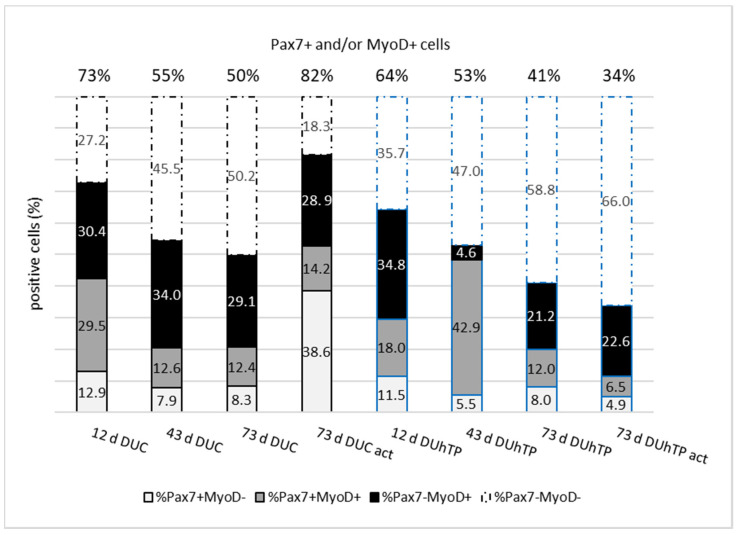
Percentage distribution of Pax7 (white), Pax7 and MyoD (gray), and MyoD (black) positive cells (Pax7+/MyoD−, Pax7+/MyoD+, and Pax7−/MyoD+) derived from sedentary male DUC (left, 12 d: *n* = 5 mice, 43 d: and 73 d: *n* = 12 mice, 73 d act: *n* = 7 mice) and DUhTP mice (right, blue border, 12 d: *n* = 8 mice, 43 d: *n* = 4 mice, 73 d: *n* = 5 mice and 73 d act: *n* = 3 mice) at the age of 12, 43, and 73 days (d) or from physically active (act) 73-day-old mice. At the top of the columns, the percentage of Pax7 and MyoD negative cells (Pax7−/MyoD−), respectively, of all considered cells, is given. For the visualization of Pax7 and MyoD, the cells were cultivated in growth-promoting conditions for three days and subsequently immunostained. At the top of the figure, the percentage of Pax7 and MyoD positive cells, respectively, of all considered cells is shown.

For more detailed characterization, isolated MPC/SATC were cultivated under growth-promoting conditions for three days and, after that, examined for expression of Pax7 and MyoD by immunostaining. While the satellite cell marker Pax7 maintains the stem cell properties of satellite cells, MyoD is expressed in proliferating cells (myoblasts) and is known to activate the transcription of genes responsible for myogenic differentiation [50]. Results are summarized in Figure 5.

Immunocytochemical staining showed an age-dependent decrease of the proportion of Pax7 and/or MyoD positive cells from 73 to 49% in DUC mice (Figure 5, upper line). This decrease resulted from a reduction of Pax7+/MyoD− and Pax7+/MyoD+ cells. However, the proportion of Pax7−/MyoD+ cells amounted to about 30% over all time points. In 73-day-old sedentary DUC mice, immunostaining revealed that 8% of cells were Pax7+/MyoD−. The voluntary activity resulted in a marked increase in the proportion of Pax7+/MyoD− cells amounting to 39% (not significant). Mice of the high-performance selected DUhTP line also showed the expected age-dependent decrease in the proportion of Pax7 and/or MyoD positive cells. Interestingly, in contrast to cell cultures derived from 12 and 73 d DUhTP mice, proliferating cells from 43-day-old DUhTP were characterized by a high proportion of Pax7+/MyoD+ cells. No significant differences were observed between cells from sedentary and exercising 73-day-old DUhTP mice, although the proportion of Pax7+/MyoD− and Pax7+/MyoD+ myogenic cells was further reduced compared to sedentary 73-day-old DUhTP mice.

However, between the lines, the proportion of Pax7 and/or MyoD positive cells was strongly decreased in active DUhTP mice (35%) compared with the control (82%). Cells from 12-day-old DUhTP mice were characterized by a reduced proportion of Pax7+/MyoD+ cells compared with DUC mice (18 vs. 30%; not significant). On day 43 of age, the percentage of Pax7+/MyoD+ cells was significantly increased in DUhTP compared to DUC mice (43 vs. 13%; *p* < 0.001), whereas the proportion of Pax7−/MyoD+ cells was decreased (5 vs. 34%; not significant).

### 3.4. MPC/SATC of the DUhTP Line Showed a Higher Ability to Fuse and to Differentiate

**Figure 6:**
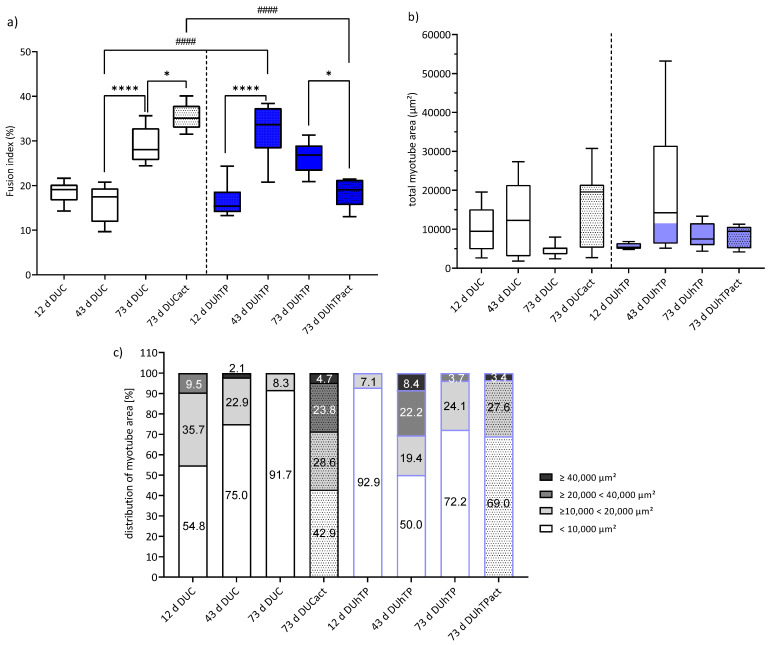
Fusion index and myotube size distribution of differentiated MPC/SATC. Myosin heavy chain immunofluorescence images were performed to quantify (**a**) the fusion index as the percentage of total nuclei being localized in fused multinucleated cells, (**b**) the area covered by myotubes (µm^2^), and (**c**) the myotube size distribution (%). All parameters are determined 72 h after induction of differentiation. Myotubes with a size of <10,000 μm^2^ were classified as small myotubes, whereas myotubes ≥10,000 <20,000 μm^2^ were grouped as middle-sized, ≥20,000 <40,000 µm^2^ as large myotubes, and ≥40,000 µm^2^ as very large myotubes. Significant line-specific differences between DUC (left; consistent of single animal and pool cultures representing: 12 d: 7 mice, 43 d: 9 mice, 73 d: 27 mice, 73 d act: 25 mice) and DUhTP (right; consistent of single animal and pool cultures representing: 12 d: 15 mice, 43 d: 25 mice, 73 d: 13 mice, 73 d act: 5 mice) lines are separated by hashtags (^####^ *p* < 0.0001). Significant age-related differences within each line are separated by asterisks (* *p* < 0.05, **** *p* < 0.0001).

After replacing the growth medium with a serum-reduced one, cells isolated from male DUC and DUhTP mice were able to differentiate and form multinucleated myotubes at all time points investigated, as clearly seen in phase-contrast images and images from MHC-stained cultures (Figure S1). The fusion index of myocytes was similar in 12- and 43-day-old DUC mice but increased significantly in cells from 73 day-old DUC mice (43 d vs. 73 d, *p* < 0.001; Figure 6a). In DUhTP mice, the fusion index of myocytes derived from muscles at day 43 was significantly higher than in 12-day-old DUhTP mice (*p* < 0.0001). In response to voluntary activity, a slightly increased fusion index in DUC mice (*p* < 0.05) and a decreased fusion index in DUhTP mice (*p* < 0.05) were found; therefore, the fusion index of the two active groups differed by 11% (*p* < 0.0001). Furthermore, myocytes of 43-day-old DUhTP mice showed a significantly higher fusion capacity than control mice of the same age (DUC: 16 ± 4% vs. DUhTP: 32 ± 4%, *p* = 0.005).

Next, as a measure of the differentiation potential, MHC-positive signals in cultures were quantified to determine the area covered by myotubes (Figure 6b, Table S1) and to analyze the myotube size (Figure 6c and Figure S1). The total myotube-covered area was highest in both mouse lines when MPC/SATC had been isolated at day 43 of life (Figure 6b, Table S1). In cell cultures derived from DUC mice, the proportion of small-sized (<10,000 µm^2^) myotubes increased in an age-dependent manner, whereas the proportion of medium-sized (≥10,000 <20,000 μm^2^) myotubes decreased (Figure 6c). Thus, the cell cultures from 73-day-old DUC mice formed mainly small-sized myotubes (92%). In response to voluntary physical activity, more middle-, large (≥20,000 <40,000 µm^2^)-, and very large-sized (≥40,000 µm^2^) myotubes were detectable (*p* < 0.04) than in sedentary littermates at the same age. Differentiated cell cultures from 12-day-old DUhTP mice consisted mainly of small-sized (93%) and some middle-sized myotubes. In cultures of 43-day-old DUhTP mice, only half of the differentiated myotubes were still of small area; the other half was defined by middle-(20%), large-(22%), and very large-area (8%) myotubes. Cell cultures from 73-day-old DUhTP mice were characterized by around 72% small-, 24% medium-, and 4% large-sized myotubes.

Comparing both lines demonstrated an increase in myotubes with a larger myotube area in DUC due to voluntary activity, while in DUhTP, these myotubes were detectable at 43 days of age (Figure 6b,c).

### 3.5. Cellular Creatine Kinase (CK) Activity

**Figure 7:**
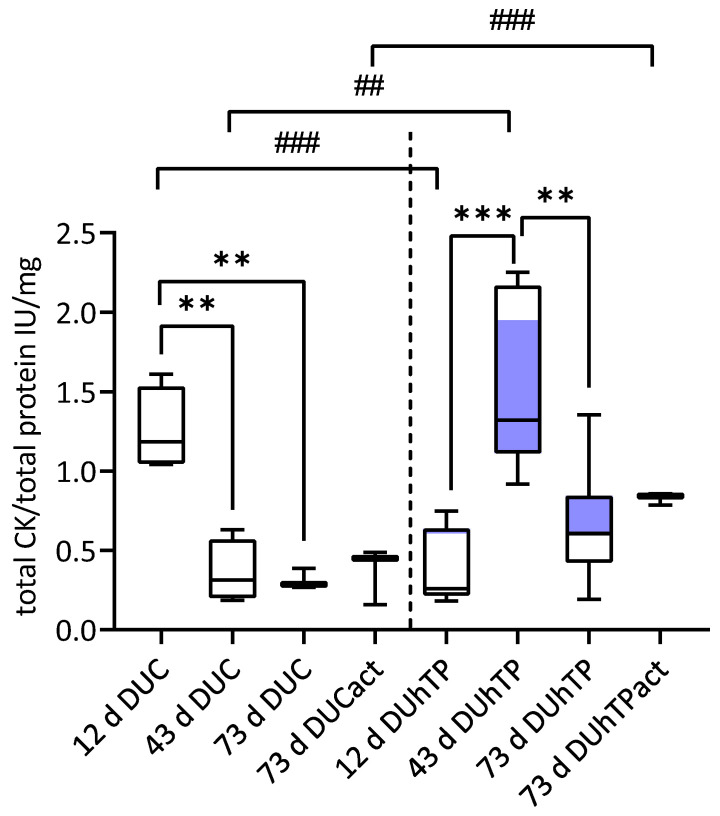
The activity of cellular creatine kinase in myotubes derived from male sedentary DUC (left, 12 d and 43 d: *n* = 4 mice, 73 d and 73 d act: *n* = 3 mice) and DUhTP mice (right, 12 d and 73 d: *n* = 7 mice, 43 d: *n* = 5 mice, and 73 d act: *n* = 3 mice) at the age of 12, 43, and 73 days (d) or physically active (act) 73-day old mice. Creatine kinase activity was determined 72 h after induction of differentiation. Results were obtained from quadruplicates, and total CK activity per total cell protein was calculated and displayed as means ± SD. Statistical significances were calculated by one-way ANOVA. Significant line-specific differences between DUC and DUhTP lines are separated by hashtags (^##^ *p* < 0.01, ^###^ *p* < 0.001), while significant age-related differences within each line are separated by asterisks (** *p* < 0.01, *** *p* < 0.001).

Next, we examined total cellular CK activity (mitochondrial and cytosolic) in myotubes after three-day cultivation in a differentiation medium. As shown in Figure 7, differentiated myotubes from DUC mice demonstrated a higher CK activity at the age of 12 days compared to the other age groups (*p* < 0.01). The highest CK activity was observed in cells of 43-day-old DUhTP mice (1.6 IU/mg total protein) compared to cells from 12-day-old (0.4 IU/mg total protein, *p* < 0.001) and 73-day-old DUhTP mice (0.6 IU/mg total protein, *p* < 0.01). Voluntary activity did not affect CK activity in both lines.

Interestingly, myotubes from 12-day-old DUC showed a higher total cellular CK activity than those from 12-day-old DUhTP mice (*p* < 0.0001). However, cells from 43-day-old and physically active 73-day-old DUhTP mice had higher CK activity than those of control mice of the same age (*p* < 0.01, *p* < 0.05, respectively).

## 4. Discussion

The present study provides data about MPC/SATC derived from male non-inbred unselected control mice (DUC) and the long-term selected marathon mouse line DUhTP established by paternal phenotypic selection for high treadmill performance without previous training. A single submaximal run on a treadmill after mating served as a selection trait and resulted in genetically fixed high running ability in the offspring [38] without increasing voluntary RW activity [40]. In the context of the superior muscle performance of DUhTP animals, muscle stem cells, namely SATC, are of particular interest.

SATC are 90% progenitor cells responsible for muscle growth and routine maintenance [5,6,7], usually by fusion with existing myofibers or with each other. In mice, SATC-dependent hypertrophic fiber growth occurs over the first three postnatal weeks [1]. As the proportion of SATC in the total muscle cell population decreases with age, falling from about 30% at birth to 3% at week seventeen [8,9,10], male mice of both lines were used at different ages to characterize SATC and their progeny in the juvenile proliferative phase (day 12), in transition (day 43), and in the “adult” phase with “quiescent” SATC (day 73). Seventy-three-day-old DUhTP and DUC mice had either access to a RW that they could use voluntarily or were kept in sedentary condition. Although the DUhTP mouse line had been selected for superior running ability, the RW measurements showed an average 2.5-fold reduction in daily voluntary activity in DUhTP mice compared to unselected controls. This difference was already observed with mice of an earlier generation but with less distinctness [40]. The covered running performance of control mice corresponded to published data with a daily RW activity between 4–20 km [51] in the RW, while DUhTP mice ran a daily distance of 2.2 km. This suggests that paternal selection for high treadmill running performance resulted in a mouse line with genetically fixed superior running performance, but that this line exerts its performance strength only under forced conditions such as on a computer-controlled treadmill. Although it is known that there exist sex-specific differences in running capacity [52,53], which we can also confirm in our mouse lines, we have limited our focus of this research to males for comparability with our previous publications.

The muscular proportion to the entire body weight was similar in both lines. However, body and muscle weight (except for day 12) were lower in sedentary DUhTP than sedentary DUC mice at all ages, as previously published for 70-day-old mice [42]. More generally, the occurrence of a small muscle phenotype, also named ‘mini muscles’, is common in various mouse lines bred for high levels of voluntary wheel running [54]. Among other changes, ‘mini muscles’ are characterized by a shift to the slower myofiber phenotype, and it is proposed that this may reduce muscle energy use, thereby providing an adaptive advantage regarding endurance-running capacity. Indeed, so far, unpublished results from a study with sedentary 70-day-old DUhTP mice confirm a higher proportion of slow muscle fibers within the musculus rectus femoris. Keefe et al. [4] found a higher number of SATC in slow myofibers of mice limb muscles (5000 per mm^3^ versus 2000 per mm^3^ in fast myofibers). In accord, our results show that compared with sedentary DUC mice, more MPC/SATC per gram muscle could be isolated from sedentary DUhTP mice, possibly indicating a higher regenerative capacity [16] and an improved ability to maintain muscle homeostasis [4].

Cultivating SATC/myoblasts from male DUC and DUhTP mice under conditions that promote proliferation reveals differences at the functional and regulatory levels. These differences manifest predominantly at day 43 of life in sedentary mice and day 73 after voluntary RW activity.

At all ages, growth profiles of myoblast cultures from DUC mice were characterized by rapid cell attachment/cell spreading (adhesion) followed by a very distinct lag phase before starting the logarithmic growth period. However, growth curves of myoblasts from sedentary 43-day-old or active 73-day-old DUhTP mice showed shortening of the lag phase and a reduced doubling time, reflecting faster activation (G0–G1 transition) and entry into the logarithmic growth period accompanied by a higher proliferation rate compared with controls. Wozniak et al. examined stretch-induced activation of SATC and identified two populations entering proliferation after different time periods (30 min and 2 h) [55]. Thus, it seems possible that SATC/MPC subpopulations with distinct activation thresholds are recruited in DUhTP vs. DUC mice. Our hypothesis is in accord with data from Rodgers et al. [56], showing the existence of two distinct functional phases, G0 and G_Alert_. In adults, SATC transition into G_Alert_ depends on mTORC1 activity and HGF receptor signaling and allows SATC to respond more rapidly to external stimuli [56,57]. In addition, proliferating cells from 43-day-old sedentary or 73-day-old active mice of the lines differ regarding their ability to attach/spread and the maximum CI. As found by others [48,58,59], a higher adhesion ability (43-day-old DUC > DUhTP; 73-day-old active DUhTP > DUC) is linked to an increased proliferation capacity of SATC descendants in our study. Thus, not only genetic background has an impact on the growth kinetics of SATC populations, but also physical exercise.

Marked differences regarding Pax7 and MyoD expression have been found in myoblast cultures from male DUC and DUhTP mice lines at these two time-points (day 43 and day 73 act) and thus, seem to be at least partly responsible for the functional changes observed.

During the postnatal development of young mice, the SATC marker Pax7 is essential for the enhanced proliferation potential, expansion, and survival of muscle progenitor cells and maintenance of their myogenic potential [5,14,15]. Moreover, during a critical period of postnatal muscle development, Pax7 is required for the transition from muscle progenitor to adult SATC status, which is induced by the withdrawal from differentiation and the transition into quiescence [16,17,60]. Thus, according to other studies [12], the proportion of Pax+/MyoD− cells was highest in day 12 SATC cultures from both lines. MyoD, on the other hand, is expressed in proliferating myoblasts and myotubes [13] and is known to force cells into the differentiation pathway by inducing the cell cycle inhibitors p21 and p57 as well as the early differentiation marker myogenin that marks the irreversible commitment to terminal differentiation [61]. Down-regulation of MyoD in a cohort of cells leads to reserve cells formation, which is important for replenishing the SATC pool [14,21,60,62,63].

It has been shown that Pax7 recognizes the entire SATC population in young mice [64] and that the subpopulation, which is MyoD negative (Pax7+/MyoD−), represents at least near ‘quiescent’ SATC, whereas Pax7+/MyoD+ cells are activated [65].

Thus, Pax7+/MyoD− cells can be considered a measure of reserve cell formation and maintenance under proliferation-promoting conditions, as in our study. Others have also identified reserve cells in in vitro culture systems and characterized them as a slowly proliferating subpopulation capable of self-renewal and generating fast-dividing progeny that undergoes terminal differentiation [6,21,59,66,67]. Hashimoto et al. (2004) investigated the reserve cell properties of slow and fast myofibers under in vitro conditions [66]. Interestingly, the process of terminal myogenic differentiation starts earlier in SATC progeny from slow myofibers, the dominating fiber type in limb muscles of DUhTP mice, and their reserve cells have a lower potential for self-renewal. Thus, along with the lower activation threshold, this gives a good explanation for the reduced proportion of Pax7+/MyoD− cells in myoblast cultures from 43-day-old sedentary or 73-day-old active DUhTP mice. Physical activity of the donor mice seems to boost this process by further stimulating activation, proliferation rate, and transition of SATC into differentiation. Under in vivo conditions, this could lead to negative effects such as reduction/exhaustion of the reserve cell pool.

In contrast, myoblast cultures from active DUC mice are characterized by a strong, 30% increase of the Pax7+/MyoD− population. This increase in SATC number is in the same order of magnitude (29% or 23%) as found by Chiarifi et al. [68] in response to endurance training (human) and by Kurosaka et al. [33] after voluntary activity in running-wheel cages (8 weeks, rat). Thus, the typical, activity-dependent enlargement of the SATC/reserve cell (Pax7+/MyoD−) compartment observed in muscles (in vivo) was also found in vitro when isolated cells from DUC mice were used. In accord with the expanded SATC pool seen in DUCact-in vitro cultures, more SATC could be isolated from limb muscles of freely active DUC mice, pointing to an increased potential for self-renewal due to activity. In addition, MPC/SATC cultures established from voluntarily active DUC mice display a higher ability to differentiate, mainly reflected by intense fusion processes and formation of more large and very large myotubes, whereas small myotubes clearly dominate in all cultures from sedentary DUC mice. In vivo, activity-induced expansion of the SATC pool and myonuclei accretion via fusion of SATC progeny have been shown to occur during hypertrophic episodes [33,34,35]. However, as described in various studies using voluntary wheel running [36,51], muscle hypertrophy was not observed in active DUC mice. On the contrary, muscle mass was reduced after voluntary RW activity. Most probably, the strength of the mechanical load was not strong enough to induce a hypertrophic muscle response in our experimental design.

Compared to active DUC mice, voluntary RW activity and the distance run were markedly reduced in active DUhTP and thus, most probably, not supportive of any significant proliferative responses in the SATC compartment. Nevertheless, it is surprising that the proportion of Pax7+ cells (Pax7+/MyoD−, Pax7+/MyoD+) is further reduced (11 vs. 20%) in active compared with sedentary 73-day-old DUhTP mice. A plausible explanation, also proofed by the existing data, could be that the majority of newly generated cells differentiate.

This idea fits the observed differences between cultures of active DUC and DUhTP mice. Active DUC mice show an increased percentage of cells positive for Pax7 and/or MyoD (82 vs. 35%), a higher fusion index (30 vs. 19%), a lower activity of CK (a marker of early differentiation; [69]), and a lower proportion of smaller myotubes than active DUhTP mice. It seems that in contrast to differentiating cultures from DUCact mice, cells from DUhTPact mice have a higher propensity to differentiate but fuse to a lesser extent with existing structures to form more mature myotubes. A deficiency in SATC cell function to supply myonuclei to myofibers accompanied by a 50% reduction of myonuclei number and a smaller fiber size were found in a viable mouse Pax7−/− model [70].

Of greatest relevance to line differences in the myoblast’s growth behavior and differentiation potential at day 43 is the ratio between Pax7 expressing (Pax7+/MyoD− and Pax7+/MyoD+) and MyoD+ cells (Pax7−/MyoD+). It amounts to 0.6 in controls and is much higher (10.5) in DUhTP animals. Specifically, the proportion of Pax+/MyoD+ cells is increased in the latter group resulting in prolonged proliferation and provision of more myonuclei. Such an effect has normally been observed after prolonged exercise [33,68,71]. Together with the higher fusion index observed in differentiating cultures from 43-day-old DUhTP mice, this might promote the formation of thicker myotubes reflective of in vitro hypertrophic processes [68]. As the proportion of Pax7−/MyoD+ cells is relatively low (5%) in sedentary 43-day-old DUhTP mice in comparison to age-matched controls (34%), it can be speculated that in controls, the committed differentiated Pax7−/MyoD+ cells persist, whereas in DUhTP mice, the transition of Pax7−/MyoD+ cells into the next steps of differentiation (upregulation of myogenin and myosin expression) is accelerated (as discussed above). This idea is supported by the significant increase in CK activity in cells isolated from sedentary 43-day-old DUhTP mice, which is known to be a useful indicator of early differentiation, also reflecting myogenin induction [69,72]. It also has been shown that the myogenic differentiation factor, MyoDl, binds specifically to two regions of the muscular CK enhancer, which is important for its in vivo expression [73]. In accord, cells isolated from sedentary 43-day-old DUhTP mice have increased proportions of Pax+/MyoD+ SATC and show upregulation of CK activity. In addition, under our in vitro conditions, a greater ability for hypertrophic growth of these cells is shown by the formation of more middle, large, and very large myotubes resulting in a higher area covered by myotubes than controls. Of note, muscular PGC-1alpha has been shown to be upregulated in treadmill-running DUhTP mice [41], and its specific isoform PGC-1alpha4 becomes activated after resistance exercise and promotes hypertrophic myofiber growth [74]. However, voluntary running wheel activity did not increase either muscular Ppargc1a1 or Ppargc1a4 expression or PGC-1alpha protein abundance [41].

Interestingly, a comparable fusion index and distribution of small, middle, large, and very large-sized myotubes were observed in differentiating cells from 73-day-old DUC mice that were physically active and sedentary 43-day-old DUhTP mice. The important role of provision of myonuclei and fusion index for robust myofiber hypertrophy has been shown in other studies [36,75,76]. Our investigation clearly shows that our selection model promotes both factors in sedentary 43-day-old DUhTP mice, but in contrast to active 73-day-old DUC mice, a cell population more primed for differentiation (Pax7+/MyoD+ vs. Pax7+/MyoD− cells in DUCact) predominates. Thus, it seems possible that besides Pax7 and/or MyoD, other genes such as calcitonin receptor, Odz4, DACH1 (a regulator of SIX and CyclinD1), Jagged-1, and CD24 known to regulate quiescence and proliferation/differentiation of SATC and their progeny are differently expressed in the lines [65,77,78]. Specifically, Odz4 has been shown to be important in maintaining the quiescence of muscle SATC and retarded the progression of myogenic differentiation [77,78]. In the study of Ishii et al. [78], Odz4-deficient mice show a reduced number and size of myofibers and reduced size of the SATC pool accompanied by accelerated activation of SATC. These results are similar to our findings with cells from active 73-day-old DUhTP mice pointing to a reduced ability to establish and maintain the quiescent state, which is a prerequisite to preserve maximum regenerative capacity [16].

## 5. Conclusions

Long-term (140 generations) paternal phenotype selection of mice for high treadmill performance (DUhTP mice) led to marked changes in the functional properties of isolated SATC and their progeny. Differences between male DUhTP and respective controls (DUC) were most prominent in 43-day-old sedentary mice and 73-day-old mice after performing voluntary wheel running for three weeks. At these time points, SATC (Pax7+ cells) from DUhTP mice are characterized by faster activation resulting in higher proliferation rates. Thus, it seems possible that due to selection, a SATC subpopulation with a lower activation threshold is preferentially recruited in male DUhTP mice.

Moreover, the area covered by myotubes and the pattern of myofiber size distribution were similar in differentiating cultures from 43-day-old sedentary DUhTP mice and those from DUC mice that were voluntarily physically active. Under both conditions, a higher proportion of medium, large, and very large myotubes were formed, indicating a robust hypertrophic response under our in vitro conditions. While this was to be expected in active animals (DUC), it shows that the selection model promotes hypertrophic processes (late fusion) in vitro without a previous activity stimulus of the donor animal. In general, the provision of myonuclei and an increased fusion index are the most important factors for a robust hypertrophic response. Consistent with this, the fusion index is high in cultures of physically active 73-day-old DUC and sedentary 43-day-old DUhTP mice. However, whereas the Pax7+/MyoD− SATC population dominates cultures from active controls, Pax+/MyoD+ cells, primed for differentiation, are most prominent in cultures from 43-day-old sedentary DUhTP mice.

Thus, it can be assumed that a combination of lower activation threshold and faster transition into differentiation (observed from day 43 on in DUhTP) could lead to negative effects such as reduction/exhaustion of the reserve cell pool under in vivo conditions. This assumption is supported by the significant reduction in Pax7 positive (Pax7+/MyoD−) cells and the reduced formation of thick fibers in cultures from active DUhTP mice.

## Data Availability

The raw data were generated at the FBN Dummerstorf. Derived data supporting the findings of this study are available from the corresponding author on request.

## Supplementary Materials

The following supporting information can be downloaded at: https://www.mdpi.com/article/10.3390/cells11061001/s1, Figure S1: Myosin heavy chain staining and contrast-phase images of differentiated myogenic progenitor cell cultures derived from 12, 43, and 73 days old sedentary and 73-day-old physically active DUC and DUhTP mice. Table S1: Myotube area (μm^2^) of differentiated myogenic progenitor cell cultures derived from 12, 43, and 73 days old sedentary DUC and DUhTP mice and active 73-day-old DUC and DUhTP mice.
