# Supplementary material for: Myogenic Precursor Cells Show Faster Activation and Enhanced Differentiation in a Male Mouse Model Selected for Advanced Endurance Exercise Performance
Source: Cells. 2022 Mar 16;11(6):1001. doi: 10.3390/cells11061001 (PMC8947336; doi:10.3390/cells11061001)
Supplement: Supplementary file 1 [file cells-11-01001-s001.zip › cells-1573415-supplementary.pdf]

Supplementary files:

Table S1: Myotube area (μm²) of differentiated myogenic progenitor cell cultures derived from 12, 43 and 73 days old sedentary and active 73-day-old DUC (left; consistent of single animal and pool cultures representing: 12d: *n* = 7 mice, 43d: *n* = 9 mice, 73d: *n* = 27 mice, 73d act: *n* = 25 mice) and DUhTP mice (right; consistent of single animal and pool cultures representing: 12d: *n* = 15 mice, 43d: *n* = 25 mice, 73d: *n* = 13 mice, 73d act: *n* = 5 mice). The values of myotube areas are expressed as means ± SD.

Age/activity groups	Myotube area (μm²)	
	DUC	DUhTP
12 d	9877 ± 6091	5582 ± 753
43 d	12,657 ± 9950	19,608 ± 18,411
73 d	4879 ± 1645	8312 ± 312
73 d act	15,322 ± 10,602	8195 ± 2969

Figure S1:

a)

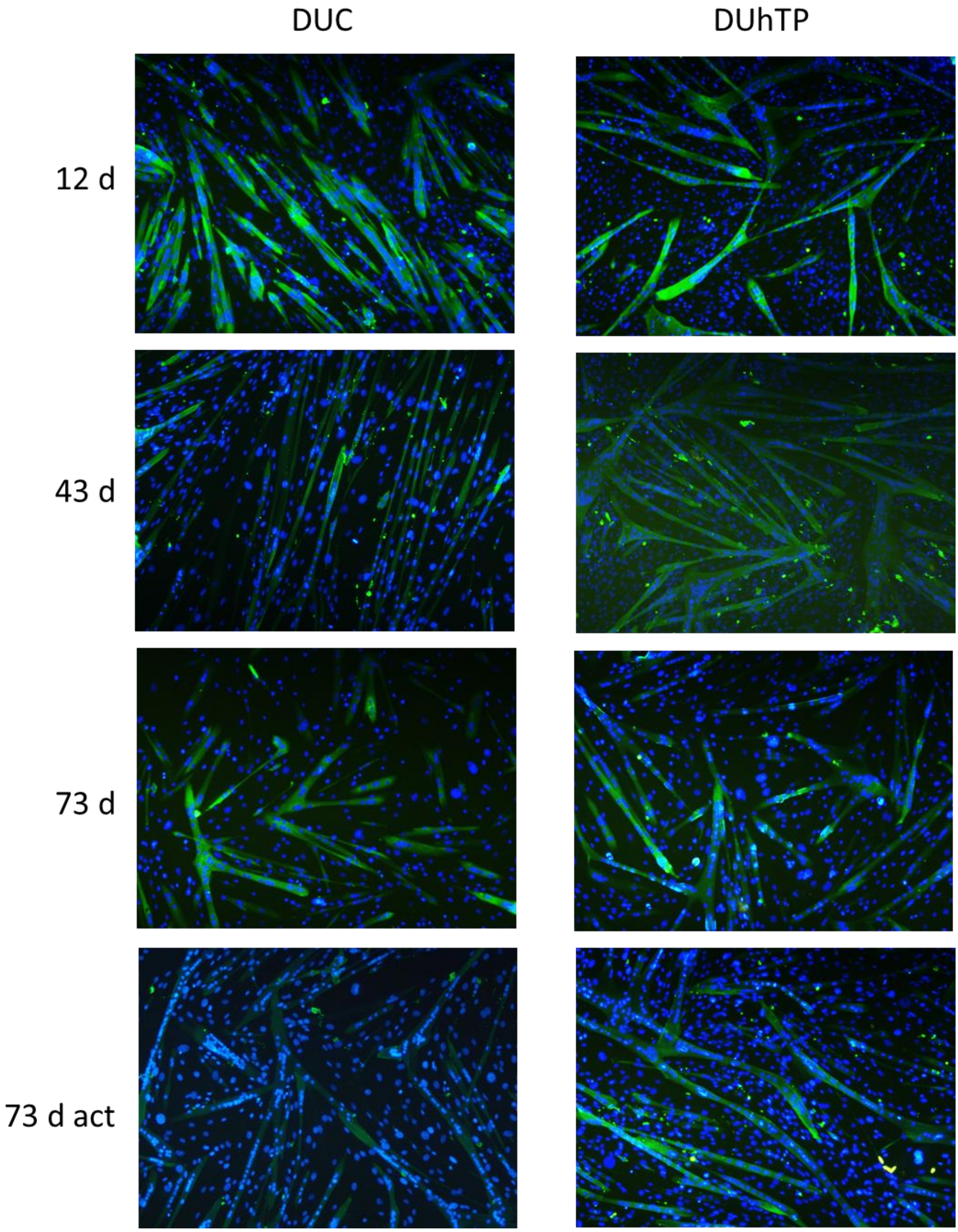

b)

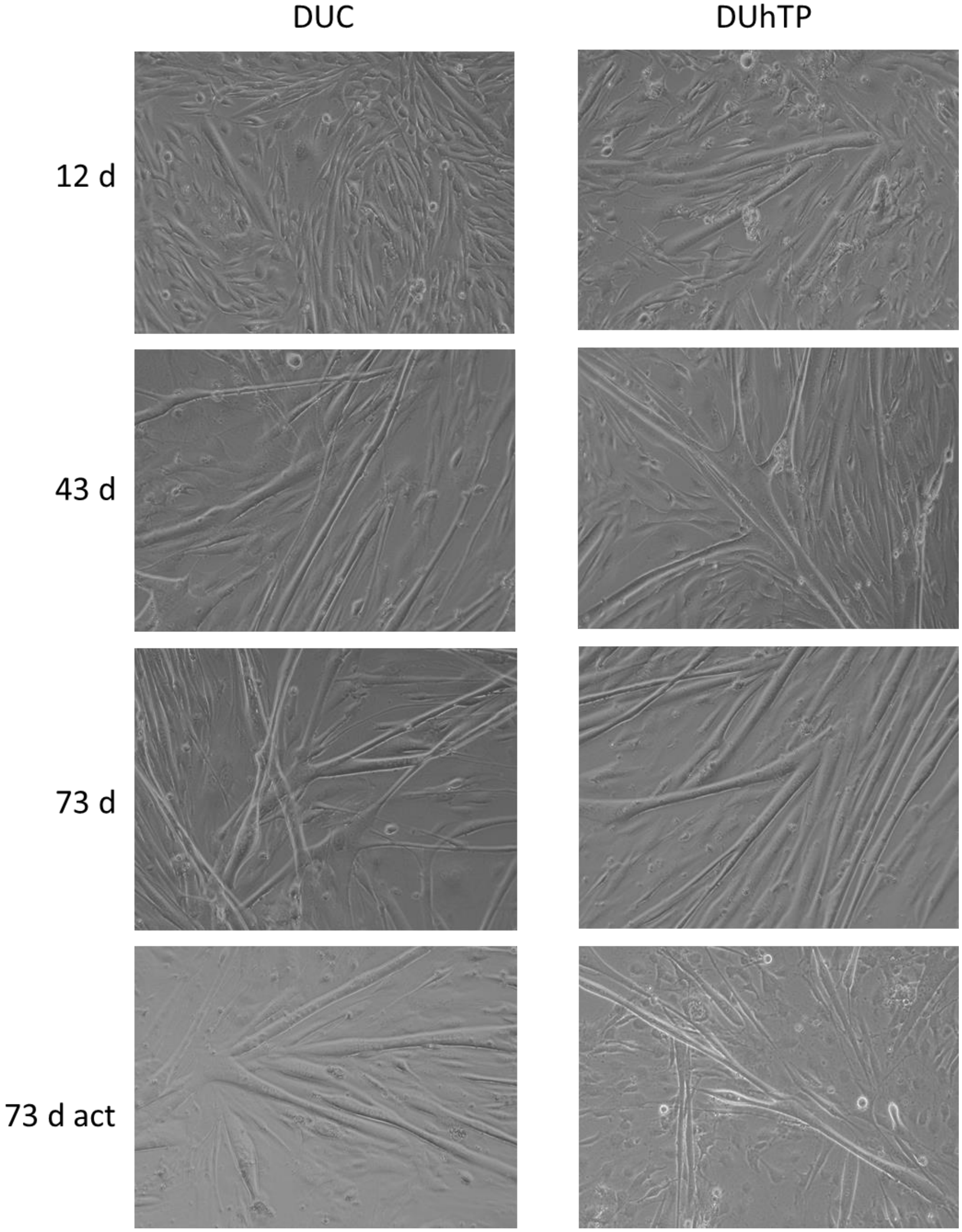

Figure S1: Myosin heavy chain staining and contrast-phase images of differentiated myogenic progenitor cell cultures derived from 12, 43, and 73 days old sedentary and 73-day-old physically active DUC and DUhTP mice. Myosin heavy chain staining and contrast-phase images of differentiated myogenic progenitor cell cultures derived from 12, 43, and 73 days old sedentary DUC and DUhTP mice and from mice (day 73) having performed voluntary activity. Differentiating cultures of myogenic precursors cells (MPC) isolated from the Dummerstorf Control mouse line (DUC, left) or the mouse line DUhTP (right) that has been selected for high treadmill performance. Representative pictures of (a) cultures immunostained for Myosin heavy chain and (b) contrast-phase images are shown for cultures derived from sedentary 12-, 43-, or 73-day-old mice, and from 73-day-old mice after performing

voluntary physical activity for three weeks. MPC from single animals or cell pools (DUC: 12 d: n = 7 mice, 43 d: n = 9 mice, 73 d: n = 27 mice, 73 d act: n = 25 mice; DUhTP: 12 d: n = 15 mice, 43 d: n = 25 mice, 73 d: n = 13 mice, 73 d act: n = 5 mice) were seeded in Matrigel-coated 24-well plates (50,000 cells/well) and first cultured in growth medium. When cells started to show signs of spontaneous differentiation, they were transferred to the differentiation medium to induce myogenic differentiation. After 72 hours in the differentiation medium, contrast-phase images were taken. Then, cells were fixed and immunostained for MHC (green) to indicate their differentiation potential; cell nuclei were stained with DAPI (blue). Micrographs of MHC-stained cultures were used to quantify the area covered by myotubes, the myofiber size distribution, and the fusion index as measures of the differentiation potential. For each experiment, six random sections were evaluated.
